# The inclusion of hyoid muscles improve moment generating capacity and dynamic simulations in musculoskeletal models of the head and neck

**DOI:** 10.1371/journal.pone.0199912

**Published:** 2018-06-28

**Authors:** Jonathan D. Mortensen, Anita N. Vasavada, Andrew S. Merryweather

**Affiliations:** 1 Department of Mechanical Engineering, University of Utah, Salt Lake City, UT, United States of America; 2 Gene and Linda Voiland School of Chemical and Bioengineering, Washington State University, Pullman, WA, United States of America; 3 Department of Integrative Physiology and Neuroscience, Washington State University, Pullman, WA, United States of America; 4 Washington Center for Muscle Biology, Washington State University, Pullman, WA, United States of America; University of Memphis, UNITED STATES

## Abstract

OpenSim musculoskeletal models of the head and neck can provide information about muscle activity and the response of the head and neck to a variety of situations. Previous models report weak flexion strength, which is partially due to lacking moment generating capacity in the upper cervical spine. Previous models have also lacked realistic hyoid muscles, which have the capability to improve flexion strength and control in the upper cervical spine. Suprahyoid and infrahyoid muscles were incorporated in an OpenSim musculoskeletal model of the head and neck. This model was based on previous OpenSim models, and now includes hyoid muscles and passive elements. The moment generating capacity of the model was tested by simulating physical experiments in the OpenSim environment. The flexor and extensor muscle strengths were scaled to match static experimental results. Models with and without hyoid muscles were used to simulate experimentally captured motions, and the need for reserve actuators was evaluated. The addition of hyoid muscles greatly increased flexion strength, and the model is the first of its kind to have realistic strength values in all directions. Less reserve actuator moment was required to simulate real motions with the addition of hyoid muscles. Several additional ways of improving flexion strength were investigated. Hyoid muscles add control and strength to OpenSim musculoskeletal models of the head and neck and improve simulations of head and neck movements.

## Introduction

Musculoskeletal modeling is a powerful tool that can be used to study human neck kinematics, dynamics, injury risk, and strength capabilities. Several models of the human neck and head have been developed, for various purposes. Dibb and Chancey and colleagues [1, 2] used models of the neck and head to investigate the effect of active muscles on neck response and injury tolerances. Models of the head and neck are often used to investigate the injury potential of automobile impacts [3–5]. De Bruijn et al. [6] recently published a detailed finite element model with 48 degrees of freedom and validated the muscle response to investigate head and neck motion during automotive impacts. A model designed for similar applications among female subjects was recently created by Östh et al. [7]. Jin et al. [8] used a finite element model of the head and neck to investigate the effect of active neck muscles during American football impacts. While these models provide knowledge about injury and failure, models of this kind are not commonly used to compute the muscle activations necessary to reproduce experimentally obtained motions. OpenSim musculoskeletal models are better suited for this purpose [9]. OpenSim models also offer a reduction of computational time required for analysis as compared to other types of models [10, 11].

While there have been many musculoskeletal models developed for the head and neck, there is a lack of models that can easily estimate muscles forces during dynamic simulations [12, 13]. Realistic musculoskeletal models of the head and neck with this function will aid in understanding the role of neck muscles during movements, with applications in ergonomics, rehabilitation, analysis of disease, and impact situations.

An OpenSim model of the head and neck was published in 2008 based on work done by Vasavada et al. [14] to explore moment generating capacities of individual muscles in various neck positions. However, this model lacks inertia properties necessary for dynamic simulation. Vasavada et al. also acknowledged that this model was not capable of producing flexion moments that are as large as have been reported in literature. This was attributed to the possibility of incorrect centers of rotation for the various joints, the lack of infrahyoid muscles, or perhaps other unknown factors.

Recently, an improved OpenSim head and neck model was published by Cazzola et al. [12] to study loading on the cervical spine during rugby. This model was verified and validated according published best practices [15]. Cazzola referred to this model as a Musculoskeletal model for the Analysis of Spinal Injuries (MASI). This model was based on Vasavada’s model, and includes inertia properties. For the MASI model, the isometric strength of each muscle in Vasavada’s model was increased by at least 40% with the aim of matching experimental data [16]. Even with stronger muscles, Cazzola reports that this model generates a flexion moment that is one-third of experimental data. The MASI model has been used for dynamic simulations, and is an important addition to musculoskeletal modeling of the head and neck.

A closer investigation of both the Vasavada and the MASI models reveal that the muscles in each model cannot adequately actuate the upper cervical spine. Both the models include constraints that reduce the degrees of freedom in the cervical spine to 6. These degrees of freedom include flexion/extension, lateral bending, and axial rotation for both the upper (skull-C1) and lower (C2-T1) cervical spine. The main flexor muscle, the Sternocleidomastoid, causes flexion in the lower cervical spine while causing extension in the upper cervical spine in these models. To overcome the inability to adequately generate flexion moment in the upper cervical spine, reserve actuators are necessary for dynamic simulation using the MASI model. Reserve actuators apply moments to actuate the model to follow experimental kinematic data, when muscles alone cannot generate sufficient moments [11, 15].

Inability to generate realistic moments in all directions is a common problem of models of the head and neck [6, 12, 14]. De Bruijn et al. [6] note that moment generating capacity of models of the head and neck are more realistic when the entire cervical spine is required to be in equilibrium. However, even with this improvement, the extension moment capacity published by de Bruijn et al. [6] was nearly 150% of experimental values, suggesting that additional improvements are necessary.

The Vasavada and MASI models measured flexion capability by comparing the maximum flexion the muscles could generate in the lower cervical spine with experimental values [12, 14]. This approach does not require equilibrium at all joints or take into account the lack of control in the upper cervical spine. These models would benefit from improvements that increase moment generating capacity and control in the upper cervical spine.

The addition of hyoid muscles to both of these models may address the limitations of weak flexion strength and adequate control in the upper cervical spine. Hyoid muscles are the group of muscles that attach to the hyoid bone, and play an important role during mastication [17]. Infrahyoid muscles, which are inferior to the hyoid bone, have been suggested to play a significant role in neck flexion [1, 18]. Siegmund et al. [19] found that infrahyoid muscles activate during flexion. Several previous models of the head and neck have included infrahyoid muscles with encouraging results [1, 2, 6–8, 20]. While these muscles are relatively small, they have large moment arms in flexion [1]. Suprahyoid muscles, which are superior to the hyoid bone, may also have a role in generating neck flexion. The location of the suprahyoid muscles may provide control for the upper cervical spine, when activated in concert with the infrahyoid and other muscles that stabilize the jaw.

The purpose of this study was to investigate the effect of hyoid muscles on moment generating capacity and dynamic simulations in an OpenSim model based on the Vasavada and MASI models. The moment generating capacity of this new model and the MASI model was computed by simulating methods found in experimental studies with the aim of requiring the entire cervical spine to be in equilibrium. The models were also evaluated by their ability to perform dynamic simulations for a variety of head movements defined through experimental motion capture.

## Methods

Three main tasks were accomplished. First a novel model, referred to as the HYOID model was created by modifying existing models to include hyoid muscles and passive elements that approximate the passive stiffness of the cervical spine. Next the moment generating capacities of the HYOID model and the MASI model were tested by simulating an isometric strength test. The strength of muscles in the HYOID model was scaled to improve moment generating capacities. The scaled HYOID model and the MASI model were used to simulate several dynamic movements specified by experimental motion capture. Joint kinematics, muscle activations, and the need for reserve actuators were compared for these dynamic simulations.

### The HYOID model

The starting point for the improved model was to combine the Vasavada and MASI models. The MASI model includes realistic inertial properties, muscles with scaled strength, a novel scapula joint, and a complete skeletal model. All joints except for those in the neck were actuated entirely by reserve actuators. As the muscles in the MASI model were scaled non-uniformly [12], only the inertial properties and joint definitions from the MASI model were used. The muscle strengths and other properties from the Vasavada model were used; however an updated muscle model to enable faster computation was used [21]. Elements based on previous studies [22, 23] that approximate passive forces through rotational stiffness and damping between each vertebral joint due to ligaments and other structures were also added (see Table 1).

**Table 1:** Properties of passive elements in HYOID model.

Joint	Ext/Flex/Lateral Stiffness (Nm/rad)	Axial Stiffness (Nm/rad)	Ext/Flex/Lateral Damping (Nm/(rad/s))	Axial Damping (Nm/(rad/s))
T1/C7	27.5	13.75	2.75	1.375
C7/C6	27.5	13.75	2.75	1.375
C6/C5	27.5	13.75	2.75	1.375
C5/C4	23.2	11.6	2.32	1.16
C4/C3	23.2	11.6	2.32	1.16
C3/C2	7.8	3.9	0.78	0.39
C2/C1	7.8	3.9	0.78	0.39
C1/Skull	7.8	3.9	0.78	0.39

**Fig 1:**
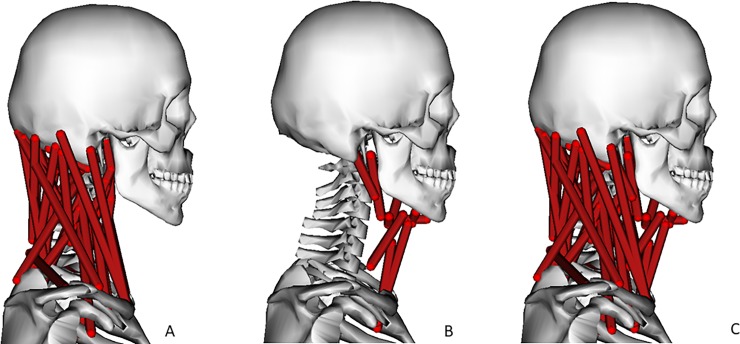
Model before addition of hyoid muscles (A), added hyoid muscles (B), all muscles in HYOID model (C).

Hyoid muscles were added based on several studies [17, 24–26]. The position and muscle parameter data for the suprahyoid muscles were obtained from Van Eijden [26]. The data for the infrahyoid muscles was obtained from Van Ee and Chancey [1, 27]. The total number of muscle actuators added to the original Vasavada model was 20 (see Table 2 and Fig 1), bringing the total number of muscle actuators in the model to 72. To simplify the model, the hyoid muscles attachment points were assumed to move with the cervical bones. The infrahyoid muscles attachment points moved with C3, which has been demonstrated to be effective in the sagittal plane [28]. The suprahyoid muscles attachment points move with C1 to avoid excessive changes in muscle length during axial rotation. The hyoid muscle maximum isometric forces were based on Vasavada’s assumption of multiplying the PCSA by 35 N/cm^2^.

**Table 2:** Properties of hyoid muscles in HYOID model. The origin of the coordinate system used is the center of the C1-Skull joint. The x-axis points anteriorly, the y-axis points superiorly, and the z-axis points to the model’s right. Each of these muscles was added bilaterally.

	Origin (m)			Insertion (m)			Max Isometric Force (N)	Optimal Length (m)	Slack Length (m)	Pennation angle at optimal (rad)
Muscle	X	Y	Z	X	Y	Z				
Sterno_hyoid	0.019	-0.172	0.020	0.050	-0.072	0.006	20.300	0.074	0.032	0.000
Omo_hyoid	0.012	-0.114	0.038	0.045	-0.063	0.013	26.250	0.046	0.020	0.000
SternoThyroid	0.018	-0.179	0.015	0.033	-0.092	0.013	22.750	0.062	0.027	0.000
digastric_post	0.016	-0.030	0.029	-0.006	0.003	0.048	40.600	0.021	0.034	0.250
digastric_ant	0.030	-0.073	0.006	0.030	-0.064	0.017	40.600	0.014	0.005	0.000
Geniohyoid	0.028	-0.069	0.005	0.069	-0.067	0.002	33.950	0.034	0.005	0.000
Mylohyoid_Post	0.032	-0.071	0.003	0.041	-0.039	0.029	18.550	0.028	0.014	0.000
Mylohoid_Ant	0.054	-0.072	-0.003	0.058	-0.060	0.016	55.650	0.020	0.003	0.000
Stylohyoid_Lat	0.021	-0.065	0.015	0.011	-0.001	0.038	6.820	0.018	0.050	0.082
Stylohyoid_Med	0.020	-0.060	0.016	0.012	-0.002	0.038	6.820	0.018	0.044	0.082

This model builds on model validation published by both Vasavada and Cazzola [12, 14]. Further exploration of this model is performed by comparing force generating capabilities determined through simulating the experiments conducted by Fice [16], and investigating the required reserve forces during motions defined by experimental motion capture.

### Model moment generating capacity

Previous studies have estimated moment generating capacities about a particular joint by summing the moment generating capacities of individual muscles about that joint [6, 12, 14]. Instead, this study estimates moment generating capacities by simulating an actual experiment. This method avoids the potential source of error from measuring moment capabilities about different joints experimentally, and the error associated with assuming the upper cervical spine can remain stable while muscles apply a maximum moment about the lower cervical spine.

**Fig 2:**
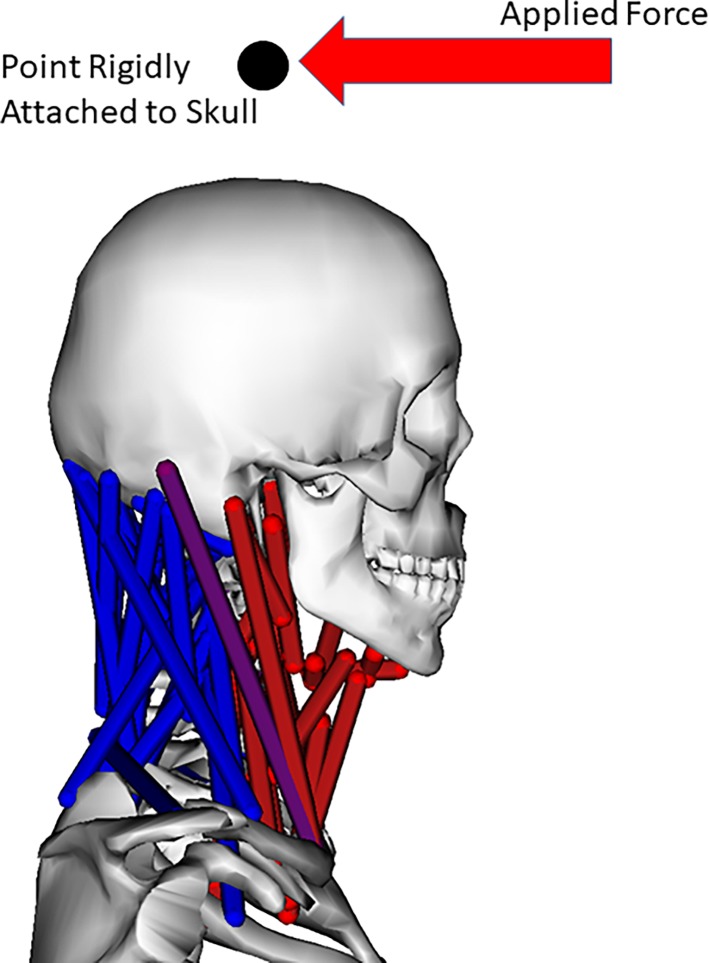
Simulation of experimental setup to compute neck moment generating capacity. Red muscles indicate active muscles. The applied force is 20 cm above the C1-Skull joint. 150 N, 200 N, 150N, and 20 Nm were applied to compute respectively the flexion, extension, lateral bending, and axial rotation moment generating capacities.

Fice et al. measured moment capabilities of the neck by instructing subjects to contract maximally while their heads were constrained by a helmet that was attached to a load cell directly above their head [16]. This experiment can be recreated in OpenSim by applying a force to the skull, with the location of the force being above the head (Fig 2).

OpenSim’s Static Optimization (SO) tool was used to determine the maximum force the model can generate in any given direction [29]. The SO tool computes muscle activations required to generate specified motions, while optimizing to minimize the sum of the all activation levels raised to a user specified value. The objective function used for optimization in the SO tool is:

$$J=\sum_{m=1}^{n}(a_{m}{)}^{p}$$ (1)

Where *n* is the number of actuators in the model, *a* is the activation level of muscle *m* and *p* is the power value specified by the user. We chose the default power value of 2, and used the default setting of including the force-length-velocity in the analysis [29]. Reserve actuators are employed when muscles alone are unable to generate the forces required for the specified motion. This is ensured by applying only a small load for each unit of activation in the reserve actuators.

First we specified the motion to be a neutral static posture. Next we applied a force to the skull in a similar position (20 cm above the C1-Skull joint) and direction as a load cell would in experiments. We also added reserve actuators in the same location and direction as the applied forces with a specification of only 1 N or 0.1Nm for every unit of activation. The SO tool then allows for computation of the force neck muscles can generate at the location of the load cell by subtracting the computed reserve actuator forces from the applied forces. Using SO instead of forward simulations provides a convenient method to replicate an isometric experimental test. Fig 2 depicts such a simulation, including which muscle are active. Fice et al. measured the distance from the load cell to the C7-T1 interface, and multiplied it by the measured forces to obtain moment generating capabilities. The same approach was taken in simulations using OpenSim by multiplying the computed force from neck muscles by the vertical distance between the applied force and the C7-T1 joint in the models (32 cm). In this way, the moment generating capabilities of the MASI and HYOID models were computed and compared to experimental data obtained from both Fice et al. and Vasadava et al. [16, 30].

The suprahyoid muscle strength was also examined. Iida et al. [31] reported that the jaw opening force in healthy adults is about 95 N. Using OpenSim’s force reporter analysis, it is possible to determine the suprahyoid muscle’s ability to generate moment at the jaw joint. This is done by performing a forward simulation with all joints locked and the suprahyoid muscles activated to maximum. The force reporter analysis in OpenSim can then be used to determine the moment generated at the jaw joint. This moment is used to calculate the jaw opening force generated by the suprahyoid muscles.

### The scaled model

Even with careful muscle modeling based on anatomical studies, scaling the strength of muscles is often required to overcome limitations in musculoskeletal models [32, 33]. This is in part due to the difficulty of choosing a realistic PCSA multiplier to find maximum isometric strength. PCSA multipliers have ranged from 35 to 137 N/cm^2^ [33]. Buchanan et al. [33] suggest that elbow flexors and extensors may have different scaling factors, which suggests that flexor and extensor muscles in the neck may also require different scaling factors. Cazzola made this assumption for the MASI model [12]. After the strength measurements were performed on the HYOID model, scaling factors for the flexion and extension muscles were chosen to increase the strength of the model to match experimental values in all directions. After the HYOID model was scaled, its moment generating capacities were measured again.

### Dynamic simulation

Using a motion capture system, kinematic data were collected for one subject doing a variety of head and neck motions. Informed written consent was obtained from this subject. Procedures for obtaining written consent and all other procedures in this study were approved by the University of Utah Institutional Review Board (IRB#00094138). The subject was a 27-year-old male, that was 5’ 11” tall and weighed 175 lbs. Nine OptiTrack Flex 3 cameras captured the motions at 100 Hz using OptiTrack’s Motive Body Software (Naturalpoint, Inc. Corvallis, OR). The subject was seated with his torso restrained in a high back chair during motion capture. Markers were placed according to OptiTrack’s Biomech 57 marker set [34]. Seven markers were used: four on the skull, one at the sternum jugular notch, and one at each acromioclavicular joint. Motions included functional movements: moving the head back and forth through flexion and extension, lateral bending, and axial rotation. Motions of bringing the head around in a circle, and three motions in which the subject was instructed to move his head randomly were also captured. This procedure was meant to capture a reasonable range of motion that the human neck can achieve during volitional movement.

The MASI and scaled HYOID models were scaled to match the subject’s size. Next, the kinematic data were used to perform inverse kinematics in the OpenSim platform for both models. The inverse kinematics tool in OpenSim seeks to minimize the distance between experimental and model markers in each frame of collected data by adjusting the 6 degrees of freedom in each model. A muscle control algorithm in OpenSim (CMC) was then used to determine muscle activations to reproduce the motions determined by inverse kinematics [11]. CMC requires reserve actuators at each degree of freedom to ensure that a solution can be found for any captured motion. The reserve actuators are used whenever the model’s muscles are incapable of producing the necessary moments to reproduce kinematics. In CMC, a cost function is used to optimize muscle activations [29]. The sum of actuator activation squared is the cost function used for CMC and is very similar to Eq (1). By setting the moment generated by reserve actuators to a value of 0.1 Nm for an activation of 1, CMC only activates reserve actuators when muscles in the model are incapable of generating the necessary moments. The reserve actuator moments for the MASI and scaled HYOID models were compared to investigate the effect of hyoid muscles during dynamic simulations.

## Results

### Model moment generating capacity

Simulating an actual experiment through the use of the SO tool resulted in smaller moment generating capacities for the MASI model than in forward simulations previously reported in the literature [12]. The moment generating capacity as determined through this method was especially small in flexion (see Table 3). It was also evident that the MASI model generates an extension moment that is comparable to values reported in the literature.

**Table 3:** Comparison of moment generating capacity resolved at the C7-T1 interface of the neck as reported in literature and as simulated by the MASI, HYOID, and Scaled HYOID models.

	Flexion (Nm)	Extension (Nm)	Lateral (Nm)	Axial (Nm)	Jaw Force (N)
Experimental Fice [16]	30 (6)	51 (11)	32 (9)	13 (5)	—
Experimental Vasavada [30]	30 (5)	52 (11)	36 (8)	15 (4)	—
Experimental Iiada [31]	—	—	—	—	95.06 (27.44)
MASI Cazzola [12]	2.37	52.87	15.04	3.06	—
HYOID	11.25	36.05	19.32	7.38	111.03
Scaled_HYOID	30.24	51.45	41.06	14.73	308.60

mean (sd)

The HYOID model was weak in all directions, but was able to generate larger moments than the MASI model in every direction except extension (Table 3). This is important, because the MASI model’s muscles are all at least 1.4 times stronger than the HYOID model. By scaling the strength of the HYOID model’s extension muscles by 1.4 and the flexion muscles by 2.7, moment generating capacities were comparable to the literature in every direction. While the lateral bending moment generating capacity was high, it is just beyond one standard deviation of the data reported by Fice and within one standard deviation of the data reported by Vasavada [30, 31].

The jaw opening forces for the HYOID model were within one standard deviation of values reported in the literature, but the jaw opening forces for the scaled Hyoid model were unrealistically high.

### Dynamic simulation

**Fig 3:**
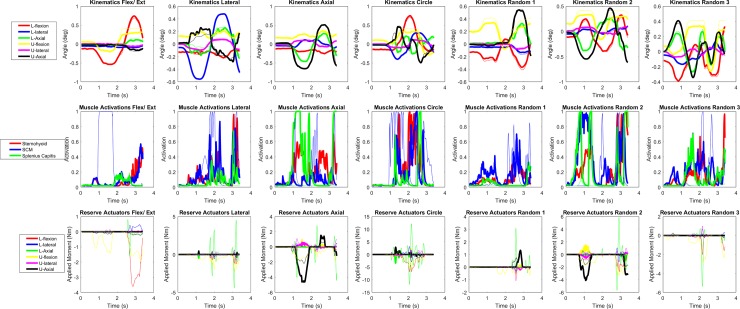
Kinematic, muscle activation, and reserve actuator data for dynamic simulations of the scaled HYOID and MASI models. All thicker lines represent the scaled HYOID model and all thinner lines represent the MASI model. “L” denotes the lower cervical spine, and “U” denotes the upper cervical spine for both the degrees of freedom and their associated reserve actuators. A negative value for flexion indicates flexion motion or moment, a positive value for lateral indicates right lateral bending, and a positive value in axial indicates left axial rotation.

The scaled HYOID model had a large reduction of reserve actuator moments in most directions compared to the MASI model (see Fig 3). A reduction in the necessary reserve actuator moments for the flexion/extension motion in the scaled HYOID model was particularly apparent (see Fig 3). Reserve actuator moments increased for axial rotations in three of the motions when using the scaled HYOID model. This trend was especially true for motions that required axial rotation of the upper cervical spine.

## Discussion

### Effect of adding hyoid muscles

The addition of hyoid muscles resulted in improvements over past models. The inability of the MASI model to generate more than 3 Nm of flexion moment when recreating physical experiments may be due to the lack of hyoid muscles. With the addition of hyoid muscles, and with every other muscle being weaker than in the MASI model, the HYOID model was able to generate more than four times the amount of flexion than the MASI model. This increase in flexion strength occurs for at least two reasons. First, the suprahyoid muscles stabilize the upper cervical spine. The sternocleidomastoid muscle causes flexion in the lower cervical spine, but also causes extension in the upper cervical spine. Without suprahyoid muscles, the model has very limited flexion moment in the upper cervical spine to resist the extension moment caused by the sternocleidomastoid. Second, the infrahyoid muscles add a significant amount of flexion moment due to their large moment arms. Even though infrahyoid muscles are comparatively small, they can contribute meaningful flexion moment simply because of their location [1].

The addition of hyoid muscles allows for more realistic simulations of the head and neck. Scaling the HYOID model’s muscle parameters allows for a large spectrum of realistic movements without the need for large reserve actuator moments. This provides a better understanding of muscle and joint reaction forces during movements of the head.

### Moment generating capacity methods

Using the SO tool as opposed to forward simulations for determining a model’s moment generating capacity has many benefits. The SO tool only allows muscle activations that result in an anteriorly directed force from the skull by minimizing an objective function (See Eq (1)). Minimizing different objective functions may result in different muscle activation patterns but cannot result in a net effect of extension of either the upper or lower cervical spine. SO ensures that the effect of active muscles on each body in the model is taken into account and can result in minimal muscle activation. Forward simulations require determining muscle activations prior to simulation. Fully activating all the flexor muscles in the MASI model results in flexion of the lower cervical spine, but extension in the upper cervical spine. The limited flexion capacity of the MASI model determined through the use of the SO tool is a result of the sternocleidomastoid muscles being inactive. SO results in these muscles being inactive to prevent excessive extension moment in the upper cervical spine. The results of this study highlight the inability of the MASI model to generate flexion in the upper cervical spine. It should be noted that the MASI model has much higher flexion moment generating capacity in scenarios that do not require flexion moment generation in the upper cervical spine[12].

### Investigating methods to improve flexion strength

In the scaled HYOID model, flexors and extensors were scaled differently (2.7 and 1.4 respectively), but scaling the flexion muscle’s strength by nearly twice as much as the extensor muscles is not biologically realistic. Unfortunately, scaling the flexion muscles by such a large factor also raises jaw opening forces to unrealistic levels. This indicates that scaling factors may not be the reason for the model being unable to produce sufficient force in flexion. Even though flexion muscles could have a different scaling factor than extensor muscles, there are several other possible reasons why the HYOID model was still weak in flexion.

Shifting the center of rotation (COR) of each vertebra in the model can have a dramatic effect in the moment generating capacities of the model. By shifting the COR for each vertebra and following the same methods as described above (see Model Moment Generating Capacity) for evaluating moment generating capacity, the effect of shifting the CORs was investigated. Shifting the COR posteriorly by 5.5 mm makes it possible to scale the strength of all muscles by the same factor, 1.8, and get realistic moment generating capacities in every direction as determined through the use of the SO tool. Doing so results in motions of the head that appear similar to motions without shifted CORs. Configuring the model into full extension with shifted CORs results in motion capture markers on the head being only 9.4 mm away from where they would be without shifted CORs. A 9.4 mm difference in head marker location for full extension may seem insignificant, but shifting the COR by 5.5 mm is not anatomically reasonable.

The COR used for the original Vasavada model were obtained from Amevo [35]. More recently, Anderst [36] showed instantaneous COR through more sophisticated and accurate techniques. Taking the average location of the COR for each vertebra from Anderst’s study results in almost the exact same results as given by Amevo. Furthermore, the 95% confidence interval of Anderst’s average COR only allows for a maximum adjustment of 1.1 mm or less. Unless the COR shifts while the spine is under load, it is unreasonable to shift the COR for these models far enough to address the flexion strength problems.

Adjusting the strength of each muscle according to more accurate data than was available to Vasavada also cannot completely account for the lack of flexion strength. The effect of changing the strength of individual muscles according to two more recent studies was also investigated. Using PCSA values from [24, 27] does not make the flexion/extension ratio reach experimental values. Using the literature to obtain updated PCSA values alone does not correct the flexion strength problem that exist in the models either.

Slight changes in posture effect the flexion and extension capabilities of the model. It is possible that the studies that measured neck strength had subjects that did not have perfectly neutral posture. The effect of posture on simulated moment generating capacity was investigated using the unscaled Hyoid model. By allowing the head to rotate 15 degrees forward, the flexion strength increases by 15% and the extension strength decreases by 5%. However, allowing for a posture change of this magnitude does not completely account for the weakness in flexion.

There are several other possible aspects of the model that could be altered to improve flexion strength. Wide muscles may need to be modeled using more actuators than exist currently in the models. Attachment points and muscle parameters such as pennation angle, optimal length, and tendon slack length may need to be updated from studies with improved methods. Modeling muscles to wrap around each other and act along a curved path may also change the flexion/extension strength ratio [37]. Yet another possibility is that raising the shoulders during exertion affects moment generating capacity of the neck. Further improvements of musculoskeletal models of the neck may require a combination of methods to improve flexion strength, and should be the focus of future studies.

### Model limitations

In addition to the limitation of flexion strength that exists in the HYOID model without disproportionate scaling of muscle strength, constraining the hyoid bone to move with cervical bones affects this model. Axial rotations require large reserve actuator forces for the HYOID model, because several of the suprahyoid muscles must be stretched significantly to allow the motion. Changing the muscle parameters to allow such stretching without supplying a resistive force would limit the need for reserve actuators, but is unrealistic. A more realistic solution would be to define the movement of the hyoid bone in three dimensions. We are unaware of any study that defines how the hyoid bone moves in three dimensions, and this should be a focus of future work to improve the HYOID model.

As stated above, this model has several limitations that may influence moment generating capability. Physiological muscles wrap around each other and interact with one another. These interactions are not modeled in the HYOID model. The upper and lower cervical bones each move as functions of the total movement of the upper and lower cervical spine, and assume a fixed COR. Allowing for independent motion of the cervical bones and moving CORs may improve results.

Despite these limitations, the HYOID model is a meaningful step forward in musculoskeletal modeling of the neck. It represents a significant improvement over previous models and is able to simulate realistic moment generation in every direction. This ability will be useful in studies that require more realistic estimates of neck strength. It has been shown that predicting strength in the principal directions accurately is a strong measure of being able to predict strength accurately in all directions [16]. The HYOID model may be useful in forward simulations of impacts to the head or torso that are likely to cause injury. These scenarios are difficult to study experimentally, so the effect of active muscles cannot be investigated without a model that can generate realistic moments without additional reserve actuators. The HYOID model should also predict accurate muscle activation patterns during motions in the sagittal plane. It is possible that scaling the extensor and flexor muscles asymmetrically could skew muscle activation patterns for axial rotation and lateral movements to favor the flexor muscles. Caution should be used in interpreting muscle activation patterns for these movements. Future studies aimed at improving flexion moment generating capacity without disproportionate muscle scaling should involve careful EMG measurements to further improve this model.

## Conclusion

Hyoid muscles improve the moment generation and movement capabilities of OpenSim musculoskeletal models of the neck. We have shown that hyoid muscles have the capability to stabilize the upper cervical spine and provide increased moment generation compared to previously published models. The HYOID model presented here simulates more realistic moment generating capacities in every direction enabling future studies in ergonomics, rehabilitation, analysis of disease, and impact situations. These studies include simulating potentially injurious events that cannot be measured experimentally, such as head impacts that occur in sports or due to slips and falls. Future studies aimed at improving the HYOID model should include experimentally measured motion of the hyoid bone in three dimensions.
